# Impact of Chronotherapy on 6-Mercaptopurine Metabolites in Inflammatory Bowel Disease: A Pilot Crossover Trial

**DOI:** 10.14309/ctg.0000000000000549

**Published:** 2022-11-22

**Authors:** Garth R. Swanson, Mary Biglin, Hannah Raff, Vijit Chouhan, Sarah Jochum, Maliha Shaikh, Lauren Francey, Faraz Bishehsari, John Hogenesch, Ali Keshavarzian

**Affiliations:** 1 Center for Integrated Microbiome and Chronobiology Research, Rush University Medical Center, Chicago, Illinois, USA;; 2 Rush University Medical Center, Department of Internal Medicine, Section of Digestive Diseases, Chicago, Illinois, USA;; 3 Divisions of Human Genetics and Immunobiology, Center for Circadian Medicine, Department of Pediatrics, Cincinnati Children's Hospital Medical Center, Cincinnati, Ohio, USA.

**Keywords:** Inflammatory bowel disease, Azathioprine, 6-mercaptopurine, Chronotherapy

## Abstract

Chronotherapy is the timing of medication according to biological rhythms of the host to optimize drug efficacy and minimize toxicity. Efficacy and myelosuppression of azathioprine/6-mercaptopurine (AZA/6-MP) are correlated with the metabolite 6-thioguanine, while the metabolite 6-methylmercaptopurine correlates with hepatotoxicity.

**METHODS:**

This was a single-center, 10-week prospective crossover trial involving 26 participants with inactive inflammatory bowel disease (IBD) on a stable dose and time of AZA or 6-MP therapy. Participants were switched to the opposite delivery time (morning or evening) for 10 weeks, and metabolite measurements were at both time points.

**RESULTS:**

In the morning vs evening dosing, 6-thioguanine levels were 225.7 ± 155.1 vs 175.0 ± 106.9 (*P* < 0.01), and 6-methylmercaptopurine levels were 825.1 ± 1,023.3 vs 2,395.3 ± 2,880.3 (*P* < 0.01), with 69% (18 out of 26) of participants had better metabolite profiles in the morning. Participants with optimal dosing in the morning had an earlier chronotype by corrected midpoint of sleep.

**DISCUSSION:**

In the first study on a potential role of chronotherapy in IBD, we found (i) morning dosing of AZA or 6-MP resulted in more optimal metabolite profiles and (ii) host chronotype could help identify one-third of patients who would benefit from evening dosing. Circadian regulation of metabolic enzymes of AZA/6-MP activity in the liver is the likely cause of these differences. This pilot study confirms the need to incorporate chronotherapy in future multicenter clinical trials on IBD disease.

## INTRODUCTION

Inflammatory bowel disease (IBD), which includes both Crohn's disease (CD) and ulcerative colitis (UC), affects 1–1.3 million people in the United States and is characterized by chronic inflammation and tissue injury of the intestinal mucosa as a consequence of inappropriate and exaggerated immune response to intestinal microbiota (1–7). Accordingly, the mainstay of IBD treatment is to control this dysregulated immune response by using medications such as immunomodulators and biologics to treat flare ups and maintain remission (8). However, state-of-the-art medical therapy with immunomodulators and biologics still fails to achieve the therapeutic goal of inducing remission and preventing flares in 40%–60% of patients (9). Emerging data suggest that optimizing the use of immunomodulators and biologics through maintaining therapeutic drug levels significantly improves the response rate (10). The current approach to achieve the therapeutic drug levels is to increase drug dosage concentration or frequency, which is often met with dose-dependent toxicity (11). Thus, an alternative approach to optimize therapeutic drug levels without increased toxicity is needed in IBD treatment.

The most commonly used immunomodulators in IBD are the thiopurines, 6-mercaptopurine (6-MP), and its prodrug, azathioprine (AZA). Clinical studies support that AZA/6-MP is effective for promoting both induction and maintenance of remission in IBD (12–15). A meta-analysis of placebo-controlled studies of patients with CD found that AZA and 6-MP were effective in inducing remission and maintaining remission in 57%–67% of patients with IBD, respectively (16). However, 30%–50% of patients with IBD may be forced to discontinue AZA and 6-MP because of side effects or a lack of efficacy of the medication (17), highlighting the need to optimize the therapeutic/toxicity ratio in AZA/6-MP therapy. AZA is a prodrug and is first converted to 6-MP in the liver. The metabolism of 6-MP is mediated by 2 enzymes, hypoxanthine guanine phosphoribosyltransferase and thiopurine methyltransferase, which result in 2 main metabolites, 6-thioguanine nucleotides (6-TG) and 6-methyl-mercaptopurine ribonucleotides (6-MMP), respectively (18,19). 6-TG, a purine antagonist, is a metabolite that interferes with DNA and protein synthesis in T cells resulting in immunosuppression and control of mucosa inflammation (20,21). 6-TG is the metabolite associated with therapeutic efficacy but also at high levels myelotoxicity, and levels above 235–260 pmol/8 × 10^8^ red blood cells have been associated with clinical remissions of IBD. On the other hand, the metabolite 6-MMP has been associated with hepatotoxicity, which typically occurs at levels greater than 5,700 pmol/8 × 10^8^ red blood cells (22). Several approaches have been tried to optimize the therapeutic/toxicity ratio of AZA/6-MP by manipulating the metabolism pathway to improve their therapeutic outcome by adding allopurinol (23) or by splitting the dose of the medication (24); however, the therapeutic outcomes of either intervention have been limited.

A new alternative approach to manipulate the 6-MP metabolism pathway is to leverage the circadian oscillation of drug metabolism pathways through time-of-day dependent dosage (“chronotherapy”). A central clock in the suprachiasmatic nucleus regulates behavioral and physiological function throughout the body in tune with the environment. The suprachiasmatic nucleus acts as a master clock that integrates environmental signals (primary light) and coordinates the activity of peripheral clocks. This leads to time-dependent responses such as the release of certain hormones, including melatonin and cortisol, which regulate proinflammatory cytokine expression and leukocyte functions, as well as oscillation in activities of enzymes that are involved in the generation of active or toxic forms of medications (25–27). An individual's chronotype is an assessment of their circadian state and diurnal preference with tendencies toward morning, evening, or intermediate (28).

Chronotherapy is the timing of medical interventions according to the host's biological rhythms to optimize drug efficacy and minimize toxicity (29,30). Although chronotherapy is not yet included commonly in medical practice, it has been shown to be beneficial in various medical treatments including hyperlipidemia, colorectal cancer, peptic ulcer disease, asthma, cardiovascular disease, and variety of chronic inflammatory disorders such as rheumatoid arthritis (31–34). The most well-studied area for chronotherapy is oncology, where toxicity from chemotherapy is frequently dose-limiting. Studies have demonstrated that changing the time of chemotherapy in diseases such as colon cancer markedly improved cancer response and decreased side effects (35). Similarly, changing the time of statin administration from morning to evening was more efficacious in decreasing low-density lipoprotein levels (36), and changing the timing of prednisone dosing to a nighttime regimen in rheumatoid arthritis resulted in decreased joint stiffness (37). Similar to rheumatoid arthritis and other autoimmune diseases, IBD is characterized by a proinflammatory cytokine upregulation, and there is significant overlap between IBD and rheumatoid arthritis treatments. Therefore, it is likely that a time-of-day intervention in medication would affect disease outcomes in IBD as well.

However, to the best of our knowledge, chronotherapy in IBD has not been studied. To fill this knowledge gap, we aimed to determine whether chronotherapy improves therapeutic drug levels in IBD. In this regard, we chose to focus on timing of AZA and 6-MP delivery because (i) the drug metabolism is well-characterized; (ii) the metabolites associated with efficacy and toxicity are well known; (iii) long-term clinical studies have been conducted that show therapeutic response and toxicity correlation with 6-TG and 6-MMP levels, respectively; and (iv) patients are maintained on once daily stable dosage of AZA or 6-MP for extended periods. Therefore, the goal of this study was to determine the chronotherapy of AZA and 6-MP in IBD that resulted in optimal metabolite levels.

## METHODS

### Participant selection

This was a single-center, Investigational Review Board-approved, prospective crossover trial involving 28 participants with inactive IBD. The study was registered on ClinicalTrials.gov NCT04304950. All participants were recruited from Rush University Medical Center IBD Clinic. The study was approved by the Institutional Review Board at Rush University Medical Center, and all patients gave written informed consent. Inclusion criteria included (i) having a diagnosis of stable UC or CD, (ii) on a stable dose and delivery time (either between 6 am and 12 pm or 6 pm and 12 am) of AZA or 6-MP therapy for at least 4 months, and (iii) no disease flares or recent steroid use over the 4 months. All participants completed baseline demographic form, blood tests, and questionnaires at their initial visit and were excluded if they met any of the following criteria: (i) clinically active disease—Harvey Bradshaw Activity Index (HBI) > 4 for CD or modified HBI > 4 for UC (38,39); (ii) baseline blood tests consistent with liver disease (liver function tests > 1.5 normal), renal impairment (creatinine > 1.5), leukopenia (WBC < 4), or 6-MMP > 10,000; or (iii) night shift, second shift, or rotating shift workers. Questionnaires included a demographic form, HBI or modified HBI, the Short-Form IBD Questionnaire (SIBDQ) (40), the Munich Chronotype Questionnaire (MCTQ), and the Morningness-Eveningness Questionnaire (MEQ). The blood test included hepatic function, white blood count, c-reactive protein, interleukin-1 (IL-1), IL-6, tumor necrosis factor-alpha (TNF-alpha), and 6-MMP and 6-TG levels. TMPT was not measured in this study, but no participants had a history of severe leukopenia or AZA/6-MP intolerance consistent with low or absent TMPT activity seen in individuals homozygous for TMPT variants.

### Protocol

Each patient was evaluated by the one of the study investigators to determine their disease activity and clinical inactivity (HBI <5 for CD or modified HBI <5 for UC). After completing the first research visit, all participants were then asked to take their AZA or 6-MP at the opposite time of day from their baseline (either between 6 am and 12 pm or 6 pm and 12 am) for 10 weeks +/− 3 days. At their second research visit, each participant had a second set of blood work and completed a second HBI or modified HBI as well as a SIBDQ. All blood work including metabolite levels was drawn in the morning (7 am–11 am) to limit the impact of time on metabolite profiles. A concomitant medication form was completed at each visit, and an adverse event log was filled out at the second visit (see the protocol in Figure 2).

### Questionnaires

The HBI determines Crohn's disease activity (41) by assessing participant sense of well-being and disease symptoms over the past day, and score >4 indicates active disease of varying severity.

The SIBDQ measures the quality of life in participants with IBD by assessing 4 quality-of-life domains: physical, social, emotional, and systemic (42). The questionnaire is scored on a 7-point Likert scale from 1 (severe problems) to 7 (no problems at all).

The MCTQ (43) and MEQ (44) are 2 validated instruments for determining participants chronotype based on sleep and wake patterns. The MCTQ contains questions pertaining to sleep and activity times, such as bedtime, length of time to fall asleep, time of awakening, and use of alarm clock on work days and work-free days. All questions are asked separately for work and work-free days. Chronotype is estimated as the midpoint of sleep on work-free days minus a correction for the difference between sleep duration on work to control for sleep debt (midpoint of sleep on work-free days, sleep-corrected) (45–47).

The MEQ consists of 19 questions regarding a participants “feeling best” rhythms and indicates preferred clock time blocks for sleep and engagement in various hypothetical situations (e.g., physical exercise, tests, and work) in addition to assessing morning alertness, morning appetite, evening tiredness, and alarm clock dependency. The MEQ score can be considered as a psychological preference for behavior, and scores range from 16 to 86, with lower scores indicating evening chronotype and higher scores indicating morning chronotype.

### Statistical analysis

All collected data, including results from blood tests and questionnaires, were kept on a REDcap database. All linear statistical analyses were performed using SPSS version 26. Circular statistics were performed on Oriana version 4. Basic summary statistics were calculated for all outcome variables of interest and pertinent covariates. The Wilcoxon signed-rank test was used for repeated measures of nonparametric data, and the Hotelling 2-sample F-test was used for comparison of the circular data.

## RESULTS

### Baseline characteristics

**Figure 1.**
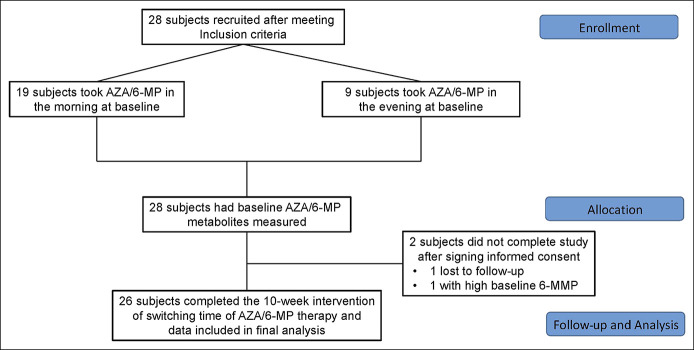
Consolidated Standards of Reporting Trials. 6-MMP, 6-methylmercaptopurine; AZA/6-MP, azathioprine/6-mercaptopurine.

A total of 28 participants were enrolled in the study, 2 participants were excluded after signing an informed consent and baseline bloodwork. One participants was unable to follow-up for the second research visit, and 1 participants was excluded because of the preintervention 6-MMP level >10,000 (Figure 1) Therefore, 26 patients successfully completed the study included in the final analysis. Of the 26 participants, 17 had CD and 9 had UC. Twelve were male and 14 were female with an average age of 53 ± 18 years. Eighteen participants (69.2%) took their medication in the morning at baseline, and 8 participants (30.8%) took their medication in the evening at baseline. Demographics are listed in Table 1. Individual participant characteristics are listed in Supplemental Table (see Supplementary Table 1, http://links.lww.com/CTG/A890).

**Figure 2.**
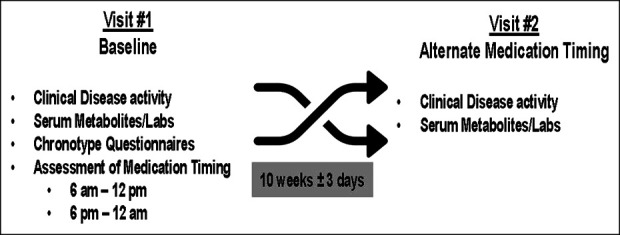
Schematic of the experimental protocol. It was determined whether participants took their azathioprine/6-mercaptopurine (AZA/6-MP) medication consistently in either an am (06:00–12:00 hours) or pm (18:00–0:00 hours) schedule. If patients did and met the other inclusion and exclusion criteria, they were invited to participate. If agreeable, after completing baseline questionnaires and blood tests, participants change the time of their medication dosing to the alternative time of day from their baseline for 10 weeks. Blood work and clinical questionnaires were then repeated.

**Table 1.** Participant characteristics

	CD (N = 18)	UC (N = 8)	*P* value
Age, median	50.2 ± 17.4	59.9 ± 21.4	0.23
Sex (male n; female n)	8:10	4:4	0.43
Ethnicity (White, non-White)	15:3	6:2	0.54
AZA:6-MP	12:6	8:0	0.13
Biologic use	12 (66%)	1 (13%)	**0.03**
AZA dose (mg/kg) 6-MP dose (mg/kg)	1.28 ± 0.54 0.73 ± 0.49	1.35 ± 0.57 N/A	N/A
Disease location	14 ileocolonic (77%)	6 pancolonic (75%)	N/A

6-MP, 6-mercaptopurine; AZA, azathioprine; CD, Crohn's disease; UC, ulcerative colitis.

### Chronotherapy and metabolite profiles

**Figure 3.**
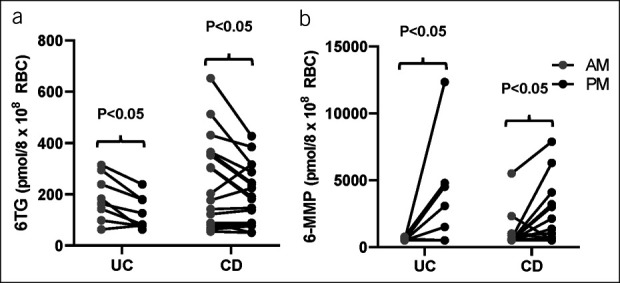
Impact of chronotherapy on azathioprine/6-mercaptopurine (AZA/6-MP) metabolites. Metabolite levels measured at 2 different time points in each inflammatory bowel disease individual, either in the am (06:00–12:00 hours) or in the pm (18:00–0:00 hours) for at least 10 weeks ± 3 days in inactive Crohn's disease (CD) and ulcerative colitis (UC). Data are shown for (a) 6-thioguanine (T-TG) and (b) 6-methyl-mercaptopurine (6-MMP). Data were analyzed by nonparametric paired analysis (Wilcoxon signed-rank test).

No participants had significant disease flares or significant side effect such as leukopenia or increased liver function tests during the 10-week intervention period where they changed the timing of the medication. The mean AM vs PM metabolite levels: 6-TG am 225.65 ± 155.05 vs pm 175.04 ± 106.89 (*P* < 0.01; paired analysis) and 6-MMP AM 825.15 ± 1,023.34 vs PM 2,395.27 ± 2,880.26 (*P* < 0.01; paired analysis), with 69% (18 out of 26) of participants having more optimal metabolite profiles in the morning (Figure 3). Specifically, of the 18 participants who took their medication in the morning at baseline, 55% (10 out of 18) had a better metabolite profile in the morning compared with the evening. Of the 8 participants who took their medication in the evening at baseline, 50% (4 out of 8) had a better metabolite profile in the morning compared with the evening.

### Host circadian rhythms compared with metabolite profiles

**Figure 4.**
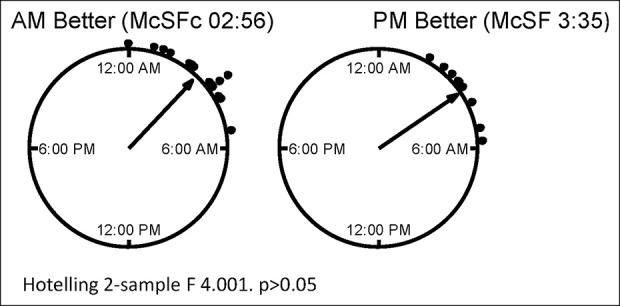
Corrected midpoint of sleep (McSF) by Munich Chronotype. Questionnaire compared with time of optimal thiopurine metabolite profile. McSF was calculated my Munich Chronotype Questionnaire at the initial visit. Optimal metabolite profiles were estimates as increased 6-thioguanine (T-TG) and lower 6-methyl-mercaptopurine (6-MMP). If no change in metabolite profile participants were excluded from analysis. The time of McSF between the 2 groups was compared by circular analysis (Hotelling 2-sample test). Azathioprine/6-mercaptopurine (AZA/6-MP).

Host chronotype as assessed by MCTQ compared with AZA or 6-MP metabolite profiles is shown in Figure 4. We found that participants with optimal dosing with more therapeutic metabolite profiles in the morning had an earlier chronotype by corrected midpoint of sleep compared with those with optimal dosing in the evening (2:34 vs 3:42; F = 4.2, *P* < 0.05).

Host chronotype as assessed by MEQ did not predict optimal metabolite profiles. Among the participants who had better metabolite level profiles in the morning vs evening, the mean MEQ score was (1.5 vs 1, *P* = 0.379).

### Circadian oscillation of metabolic enzymes

**Figure 5.**
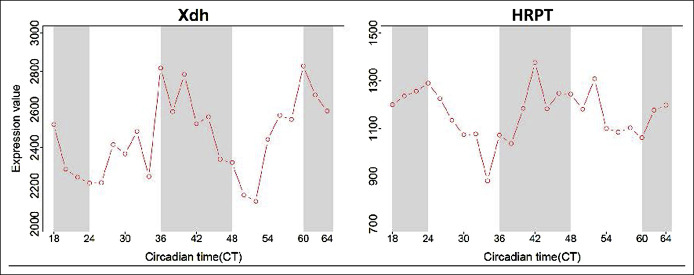
Circadian oscillation of metabolic enzymes. Using publicly available data from the Circadian Expression Profiles Database (http://circadb.hogeneschlab.org/), we examined whether metabolic enzymes of azathioprine/6-mercaptopurine (AZA/6-MP) were under circadian regulation. Several key enzymes in the metabolic pathway such as hypoxanthine guanine phosphoribosyl transferase (HRPT) and xanthine dehydrogenase (Xdh) had robust circadian oscillation based on time of day.

Using publicly available data, we examined whether metabolic enzymes of AZA/6-MP were under circadian regulation. Several key enzymes in the metabolic pathway such as hypoxanthine guanine phosphoribosyl transferase and xanthine dehydrogenase had robust circadian oscillation based on time of day. Hypoxanthine guanine phosphoribosyltransferase activity had a period of 24.0 with a q-value < 0.01, and xanthine dehydrogenase had a period of 25.0 with a q-value < 0.01 (Figure 5). These data were derived from the Circadian Expression Profiles Database (http://circadb.hogeneschlab.org/) and were in mouse liver (48).

### Impact of chronotherapy on clinical disease activity, quality of life, inflammatory cytokines, and adverse events

There was not a significant difference in clinical disease activity or quality-of-life measures with chronotherapy intervention. The mean am vs pm HBI scores were 3.09 ± 2.71 vs 2.15 ± 1.71, *P* = 0.17, respectively. Similarly, the mean preintervention vs postintervention IBDQ scores were 5.91 ± 0.63 vs 5.83 ± 0.56, respectively, *P* = 0.445.

Inflammatory cytokines TNF-α and IL-6 also did not differ significantly by am or pm dosing. TNF-α levels (pg/mL) were 1.70 ± 2.5 vs 1.59 ± 0.90, *P* = 0.49, while IL-6 levels (pg/mL) were 1.32 ± 1.68 vs 1.46 ± 0.89, *P* = 0.71.

Adverse events encountered by each participant over the course of intervention period did not differ with change of timing of the medication. Most side effects were mild to moderate and required no intervention. There were no serious adverse events that required participant discontinuation from the study.

## DISCUSSION

The primary aim of our study was to determine whether a simple intervention of changing drug delivery times of AZA and 6-MP would affect key metabolite levels that are clearly associated with efficacy and side effects. A secondary aim of this study was to determine whether these outcomes were dependent on host's circadian rhythm. The reason we selected AZA/6-MP for chronotherapy in IBD was the metabolites 6-TG and 6-MMP which are well-established and clearly associated with clinical outcomes. Indeed, we found that morning delivery of AZA and 6-MP resulted in optimizing metabolite profiles with a statistically significant increase in 6-TG levels and decrease in 6-MMP levels by paired analysis. In addition, host chronotype did affect the optimal time of therapy, with a late host chronotype being associated with more optimal metabolite levels during the evening dosage.

We did not find that chronotherapy of AZA/6-MP significantly affected clinical outcomes—HBI or SIBDQ or markers of subclinical inflammation (serum cytokines TNF-α and IL-6). This is not surprising because this study had a short follow-up (10 weeks) and was not powered to find a clinical difference. Furthermore, it included only an inactive group of participants with IBD who were on a stable dose of medication and had no recent flares in the last 4 months making detecting a clinical difference or difference in subclinical inflammation very unlikely.

Overall, our approach of increasing 6-TG level through optimizing time of drug delivery (i.e., chronotherapy) with AZA/6-MP should be preferred compared with simply increasing the dose of AZA/6-MP. This is because increasing the dose of AZA/6-MP will increase the 6 TG level but will also be associated with a higher level of toxic metabolites, such as 6-MMP. Furthermore, this strategy would avoid dose-dependent side effects of AZA/6-MP, such as nausea, dyspepsia, hair loss, and muscle pain (49). Meanwhile, the chronotherapy approach in this study was able to simultaneously increase 6-TG levels while decreasing the 6-MMP levels without increasing the dose of the medication, which could have important advantages over other treatment strategies, such as increasing the dose, splitting the dose, or adding allopurinol (17). As demonstrated in our study, morning dosing of AZA or 6-MP results in overall more optimal metabolite profiles in almost two-thirds of patients with IBD, and evaluating the host chronotype can identify most of the patients where PM dosing would be optimal. This could be of significant importance in patients with aggressive and complicated IBD because we recently found that patients with complicated IBD frequently have a late chronotype, which can affect disease course and disease activity and require step up to biologic therapy (50). Thus, in those complicated patients with IBD, optimal timing of AZA/6-MP may depend on the host chronotype, which can be performed by a simple questionnaire (MCTQ) in the clinic (51).

To our knowledge, there have been no previous studies evaluating chronotherapy in IBD. Circadian rhythms are known to control various functions in the gastrointestinal tract, including intestinal barrier function, gastrointestinal motility, and the secretion of mucus and digestive enzymes (52–56). Immune function is also under circadian regulation, with proinflammatory mediators typically elevated mainly at night as demonstrated in studies investigating chronotherapy with prednisone in rheumatoid arthritis (31). However, there is a paucity of studies investigating chronotherapy with IBD-related medications. It is important to note that in our study, host chronotype as measured by MCTQ but not MEQ was significantly associated with optimal 6-MP or AZA timing. Chronotype can be measured by MEQ or MCTQ which have both been associated with the gold standard measurement for central circadian timing and dim light melatonin onset (DLMO); however, midpoint of sleep on work-free days, sleep-corrected from MCTQ has been found to be a stronger predictor of DLMO compared with MEQ (57), which is what we found in this study as well.

In addition to assessing the central circadian clock or chronotype, the time of food consumption which is the main entertainer or zeitgeber of the peripheral clock in the gastrointestinal tract may also be important. Although we did not access the peripheral circadian clock in this study, we did recently develop a food timing questionnaire for this purpose, which could be used as a covariate in future studies (58). The impact of chronotherapy on IBD medications in IBD, whether accessing the central or peripheral gastrointestinal circadian clock, will be of increasing importance in IBD treatment. For example, several oral small molecule once daily medications have been recently approved by the US Food and Drug Administration for the treatment of IBD, including tofacitinib extended release, ozanimod, and upadacitinib (59). Determining optimal chronotherapy of small molecule therapies in IBD will be essential to improve efficacy and limit side effects.

There were several limitations in this study. First, this was a single-center study with an overall limited number of participants with IBD that focused on metabolite levels and not clinical efficacy. As the first study conducted on chronotherapy of AZA and 6-MP, this was a “proof-of-concept” pilot study, and larger multicenter studies are needed to confirm the importance of chronotherapy of AZA/6-MP on clinical end points such as mucosal disease activity. Second, the overall dose of AZA/6-MP used in this study was low because it was frequently used in combination with a biologic for its immunogenicity properties. Therefore, the dose of AZA or 6-MP was infrequently increased to the dosage typical of monotherapy (2.0 mg/kg for AZA or 1.5 mg/kg for 6-MP). Third, the study design relied on patient compliance with medication timing and did not use a medication monitoring system to ensure compliance with the intervention. Finally, circadian rhythms were assessed only by a questionnaire (MCTQ and MEQ) and not directly measured in the laboratory by DLMO.

In conclusion, chronotherapy of AZA/6-MP can optimize metabolite levels associated with increased efficacy and decreased side effects without changing the dose of the medication. In addition, host chronotype can help to determine the optimal timing for AZA or 6-MP therapy. Recently, there has been an increased understanding of the importance of incorporating a fourth dimension in medicine–time. In future IBD studies, the time of IBD medications needs to be carefully monitored along with the host circadian rhythms to achieve the goals of personalized medicine and to optimize medical therapy for each patient.

## CONFLICTS OF INTEREST

**Guarantor of the article:** Garth Swanson, MD, MS.

**Specific author contributions:** G.R.S.: hypothesis generation, study design, participant recruitment, data analysis, interpretation, and manuscript generation; M.B., H.R., and V.C.: participant recruitment, data collection, and analysis; S.J.: data analysis and data interpretation; L.F. and J.H.: data analysis, interpretation, and manuscript writing. F.B. and A.K.: hypothesis generation, study design, and manuscript writing. All authors reviewed the manuscript and revised it as necessary.

**Financial support:** This research was supported by the National Institute of Diabetes and Digestive and Kidney Diseases and the Alcohol Abuse and Alcoholism of the National Institutes of Health in part under Grant Nos. R01DK128085 (PI G.R.S.) and R24AA026801 (PI A.K.). We thank the Larry Field, Glass, Keehn, Sklar, and Alvin Baum Family fund for their philanthropic funding.

**Potential competing interests:** None to report.

**Clinical trials:** This study was registered under ClinicalTrials.gov NCT0430495.

**IRB:** This study was approved by the Rush University Investigational Review Board.

**Study Highlights**

**WHAT IS KNOWN**

✓ Drug metabolism is affected by the circadian clock.
✓ Chronotherapy is tailoring timing of medications to optimizing efficacy and minimizing side effects.
✓ Thiopurines in inflammatory bowel disease are frequently not tolerated and have known metabolites that relate to efficacy and side effects.

**WHAT IS NEW HERE**

✓ This pilot study showed that optimizing timing of thiopurines in inflammatory bowel disease resulted in a significantly improved metabolite profile that would lead to improved efficacy while limiting adverse effects.
✓ The host chronotype can be used to help predict the optimal timing of thiopurines in inflammatory bowel disease.
✓ The time a once daily medication is taken can have a significant impact on thiopurine metabolites in inflammatory bowel disease and should be individualized to improve tolerability and efficacy.

## Supplementary Material

SUPPLEMENTARY MATERIAL
