# Supplementary material for: Impact of Chronotherapy on 6-Mercaptopurine Metabolites in Inflammatory Bowel Disease: A Pilot Crossover Trial
Source: Clin Transl Gastroenterol. 2022 Nov 22;14(2):e00549. doi: 10.14309/ctg.0000000000000549 (PMC9945554; doi:10.14309/ctg.0000000000000549)
Supplement: SUPPLEMENTARY MATERIAL [file ct9-14-e00549-s001.docx]

**Supplemental Table 1: Individual Subject Characteristics**

| **Subject ID** | **Gender** | **Age** | **Race** | **UC or CD** | **AZA or 6-MP** | **Dose** | **TPMT Activity** | **Baseline AZA or 6-MP time** | **Intervention AZA or 6-MP time** |
| --- | --- | --- | --- | --- | --- | --- | --- | --- | --- |
| 1 | M | 36 | C | CD | AZA | 2 mg/kg | unknown | am | pm |
| 2 | M | 28 | C | CD | AZA | 2 mg/kg | normal | am | pm |
| 3 | MM | 32 | C | UC | AZA | 1.3 mg/kg | unknown | pm | am |
| 4 | F | 75 | C | UC | AZA | 1.5 mg/kg | unknown | am | pm |
| 5 | M | 29 | Asian | CD | AZA | 1 mg/kg | unknown | pm | am |
| 6 | F | 66 | C | CD | AZA | 1.2 mg/kg | unknown | am | pm |
| 7 | F | 66 | C | CD | 6-MP | 0.5 mg/kg | unknown | am | pm |
| 8 | M | 33 | AA | CD | AZA | 0.8 mg/kg | unknown | pm | am |
| 9 | F | 3 | AA | CD | AZA | 1.5 mg/kg | unknown | pm | am |
| 10 | M | 24 | C | UC | AZA | 1.8 mg/kg | unknown | am | pm |
| 11 | F | 35 | C | CD | AZA | 2 mg/kg | unknown | pm | am |
| 12 | M | 64 | C | UC | AZA | 0.6 mg/kg | unknown | am | pm |
| 13 | F | 58 | C | CD | AZA | 1.5 mg/kg | unknown | am | pm |
| 14 | F | 85 | AA | UC | AZA | 2 mg/kg | normal | am | pm |
| 15 | M | 40 | C | CD | AZA | 0.6 mg/kg | unknown | am | pm |
| 16 | F | 71 | C | CD | 6-MP | 0.4 mg/kg | unknown | am | pm |
| 17 | M | 70 | C | UC | AZA | 2 mg/kg | unknown | am | pm |
| 18 | F | 50 | C | CD | 6-MP | 1.5 mg/kg | unknown | am | pm |
| 19 | M | 79 | C | CD | AZA | 0.7 mg/kg | unknown | pm | am |
| 20 | M | 30 | C | CD | 6-MP | 1.2 mg/kg | unknown | pm | am |
| 21 | F | 72 | AA | UC | AZA | 0.6 mg/kg | normal | am | pm |
| 22 | M | 52 | C | CD | 6-MP | 0.3 mg/kg | unknown | pm | am |
| 23 | F | 71 | C | CD | AZA | 1.5 mg/kg | unknown | am | pm |
| 24 | F | 69 | C | CD | 6-MP | 0.5 mg/kg | unknown | am | pm |
| 25 | F | 57 | C | UC | AZA | 1 mg/kg | unknown | am | pm |
| 26 | F | 57 | C | CD | AZA | 0.6 mg/kg | unknown | am | pm |
